# Viscoelastic Slider Blocks as a Model for a Seismogenic Fault

**DOI:** 10.3390/e25101419

**Published:** 2023-10-06

**Authors:** Charlotte A. Motuzas, Robert Shcherbakov

**Affiliations:** 1 Department of Earth Sciences, Western University, London, ON N6A 5B7, Canada; 2 Department of Physics and Astronomy, Western University, London, ON N6A 3K7, Canada

**Keywords:** earthquake physics, ETAS model, friction, viscoelasticity, slider-block model, power–law

## Abstract

In this work, a model is proposed to examine the role of viscoelasticity in the generation of simulated earthquake-like events. This model serves to investigate how nonlinear processes in the Earth’s crust affect the triggering and decay patterns of earthquake sequences. These synthetic earthquake events are numerically simulated using a slider-block model containing viscoelastic standard linear solid (SLS) elements to reproduce the dynamics of an earthquake fault. The simulated system exhibits elements of self-organized criticality, and results in the generation of avalanches that behave similarly to naturally occurring seismic events. The model behavior is analyzed using the Epidemic-Type Aftershock Sequence (ETAS) model, which suitably represents the observed triggering and decay patterns; however, parameter estimates deviate from those resulting from natural aftershock sequences. Simulated aftershock sequences from this model are characterized by slightly larger *p*-values, indicating a faster-than-normal decay of aftershock rates within the system. The ETAS fit, along with realistic simulated frequency-size distributions, supports the inclusion of viscoelastic rheology to model the seismogenic fault dynamics.

## 1. Introduction

Despite the complex nature of earthquake dynamics, simple models may be used to understand many aspects of earthquake behavior. In particular, these models aim to explain and represent the physical mechanisms behind the generation of earthquakes [1,2]. This is accomplished by analyzing and replicating patterns observed in aftershock sequences and other seismic activity in accordance with statistical observations and known properties of the Earth’s crust. These models are designed to examine earthquake behavior from the perspective of either statistical seismology or physics, and allow for a more comprehensive understanding of the physics involved with common earthquake patterns [3,4]. A better understanding of these characteristics provides additional resources for risk estimation and forecasting efforts, which serve to mitigate the damage resulting from future earthquake events [5,6,7,8].

When considering a traditional earthquake model, earthquakes result from interactions between tectonic plates within the Earth’s crust. This system, defined by networks of constantly moving plates and their corresponding dynamics, can be classified as an incredibly complex nonlinear system, exhibiting self-organized criticality [9,10]. Plainly, as the plates are constantly driven forward and interacting with one another, the system hovers at a state extremely close to instability, and at a certain stress threshold, critical instabilities appear in the form of abrupt slippage along a fault [3,11,12]. In this context, earthquakes are viewed as the outward expression of these sudden slippages, as segments of the rock walls on either side of the fault are suddenly displaced in a series of “avalanches” [12,13]. The resulting earthquake dynamics within a certain region (a single fault or a system of faults) then depend on the physical properties of the surrounding rock medium, particularly its elastic, viscous, and frictional responses.

Due to the complexity of this system, it is impossible to design a model that represents the Earth’s crust in its entirety. Instead, models are designed to be much simpler analogs, which still exhibit similar system dynamics on a much smaller scale. The slider-block model, first introduced by Burridge and Knopoff in 1967, is an example of one such model used to represent the behavior of a single seismogenic fault within a surrounding elastic medium [14]. This slider-block model focuses on the interaction between two opposing walls of a fault, and how the corresponding friction and elastic responses play a role in the occurrence of earthquakes [11,12,14,15]. The original formulation represents a chain of blocks of equal mass, connected in series using elastic springs. These blocks are placed on a surface with uniform friction, which acts in opposition to a constant driving force. This system, much like the Earth’s system of tectonic plates, is persistently driven toward instability. This instability results from the constant driving force, alongside the linear and nonlinear forces acting on each block due to the elastic springs and velocity weakening friction force [12,16]. Each block eventually approaches critical slipping points, resulting in abrupt displacements of previously stuck blocks, or “avalanches” affecting a series of neighboring blocks, mimicking earthquakes propagation along a fault.

Many studies incorporating this slider-block concept have been carried out, utilizing two-dimensional arrays of slider blocks, cellular automata, and a variety of nonlinear velocity-dependant friction laws, all in an attempt to discern more information regarding the dynamics of seismogenic faults [1,3,17,18,19]. Those studies examined the sizes of generated avalanche events, and determined that models similar to the Burridge and Knopoff model produce size distributions similar to those commonly associated with seismic activity. In particular, for slider-block models and other models that display aspects of self-organized criticality, frequency-size distributions often follow power–law-type functional form with relevant finite-size effects. [1,12]. It should be stated that although slider-block models are not considered as systems that exhibit strictly self-organized critical behaviour, due to the presence of tuning parameters, these models still provide meaningful methods through which this behavior can be studied.

Despite the simple premise, the slider-block model can be a useful tool for simulating the behavior of earthquake faults as complex, chaotic systems, while simultaneously examining the properties of self-organized criticality within the field of seismology [2,3,20]. However, this model does have its limitations. In particular, large earthquakes are often followed by a series of aftershocks [21,22], proportional to the initial shock, which decrease in magnitude and frequency according to several well-studied patterns, such as the Omori–Utsu law [23], or the Epidemic-Type Aftershock Sequence (ETAS) model [24,25]. Purely elastic slider-block models often lack these aftershock patterns, reducing the applicability of these models to real-life earthquake forecasting or risk assessments. It is because of this that recent studies have introduced slider-block models with additional components or processes to better resemble seismologic observations, primarily through the introduction of physical properties such as viscoelasticity [19,26,27,28,29,30,31]. The addition of viscoelastic components to existing slider-block models serves to recreate the physical properties of the Earth’s crust along a seismogenic fault, in the hopes of recreating realistic aftershock sequences following a sufficiently large event.

Although the linearly elastic properties of a rock medium strongly influence earthquake dynamics over short timescales, nonlinear properties of the Earth’s crust are thought to influence many aspects of observed seismic activity [32,33]. The lithosphere, the lower regions of the Earth’s crust, the upper mantle, and regions along active fault zones exhibit the greatest deviation from linear elasticity [34,35,36,37]. In these regions, the addition of nonlinear viscoelasticity may be responsible for the presence of the temporal clustering, or aftershocks, generated independently of the initial driving forces behind the initial avalanche [19,28,38].

In this paper, we investigate the influence of viscoelasticity on a one-dimensional slider-block model, specifically through the use of standard linear solid (SLS) viscoelastic components, composed of a Maxwell element connected in parallel to an elastic spring. Using computer simulation to depict the motion of each slider-block over a set time interval, a catalog of avalanche events is then analyzed using the ETAS model to determine the model parameters and the quantitative behaviour of the model. The viscoelastic slider-block model reproduces frequency–magnitude behavior and temporal clustering similar to that of natural seismic activity. Reasonable values are obtained through ETAS parameter estimates, which indicate the generation of realistic, rapidly decaying aftershock sequences. The purpose of this model is to investigate the behavior of simulated earthquake catalogs. This is performed to better understand the influence of different physical properties on seismic activity, using metrics like ETAS model parameters to compare simulated events to real-life seismicity.

The paper is organized as follows. In Section 2, the model is formulated and the governing equations are derived. In Section 3, the model simulations are presented and the obtained results are described. And finally, in Section 4, the results are discussed and future research directions are outlined.

## 2. Viscoelastic Slider-Block Model

**Figure 1:**
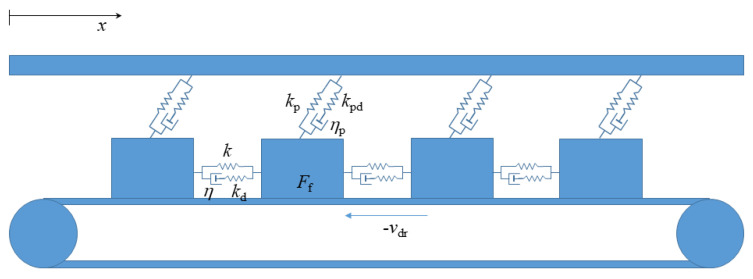
Schematic illustration of the model with N=4 blocks, with model parameters $\left\{k,k_{d},\eta ,k_{p},k_{\mathrm{pd}},\eta_{p}\right\}$. The slider blocks are interconnected by SLS elements and are driven by the conveyor belt. The positive *x* direction is opposite the direction of the driving velocity, $-v_{dr}$.

The model consists of a one-dimensional chain of slider blocks, each of equal mass *m* and connected to neighboring blocks by the SLS elements. This model is illustrated in Figure 1. These SLS components contain a viscous dashpot and an elastic spring, with characteristic parameters η and $k_{d}$, respectively, connected in series. This is known as a Maxwell element. This Maxwell element is connected in parallel to a second elastic spring, with parameter *k*, to form the SLS component [39]. This component was chosen to model the observed nonlinear viscoelastic response of common natural materials, specifically with regard to the behavior of the Earth’s crust. Each block is then individually attached to an upper plate via separate SLS components, characterized by parameters $k_{\mathrm{pd}}$ and $\eta_{p}$ within the Maxwell element, and parameter $k_{p}$ for the elastic spring placed in parallel. The chain of blocks is then placed on a conveyor belt, which moves at a constant driving velocity, $-v_{\mathrm{dr}}$.

This model also relies on a nonlinear slip–stick friction law, dependant on the velocity of the blocks relative to the moving conveyor belt. The chosen friction law originates from the 1989 Carlson and Langer slider-block model, in which the magnitude of the friction force ranges between $f_{s}$ and $-f_{s}$. This friction force takes the form [11]:

(1) $$F_{f}\left(v_{i}\right)=f_{s}\phi \left(\frac{v_{i}+v_{dr}}{v_{0}}\right),$$

where $v_{i}$ is the velocity of a single block *i*; $v_{0}$ is a chosen reference velocity; and the velocity-dependant component, ϕ, is chosen such that the friction force vanishes at high velocity. In this model, ϕ is defined as follows [11]:

(2) $$\phi \left(z\right)=\left\{\begin{array}{cc} (-\infty ;1]\;, & z=0\;,\\ \frac{\mathrm{sign}(z)}{1+\delta |z|}\;, & z>0\;. \end{array}\right.$$

To simulate the motion of *N* blocks in a linear array, the equation of motion for a single block *i* can be written as

(3) $$\begin{array}{c} m\frac{d^{2}x_{i}}{dt^{2}}=-k\;\left(2x_{i}-x_{i-1}-x_{i+1}\right)-k_{p}\;x_{i}+F_{M}^{\left(i-1\right)}\left(t\right)+F_{M}^{\left(i+1\right)}\left(t\right)\\ +F_{\mathrm{Mp}}\left(t\right)-f_{s}\;\phi \left(\frac{v_{i}+v_{\mathrm{dr}}}{v_{0}}\right)\;, \end{array}$$

where $F_{\mathrm{Mp}}\left(t\right)$ is the force exerted by the Maxwell element connecting the block to the top plate, and $F_{M}^{\left(i-1\right)}\left(t\right)$ and $F_{M}^{\left(i+1\right)}\left(t\right)$ are the forces due to the two Maxwell elements connecting the block to its neighboring blocks. When the forces acting on the block from both the upper driving plate and the nearest neighbor blocks exceed that of the velocity-weakening frictional force, the block abruptly slips. These sudden displacements may trigger subsequent slippages of neighboring blocks, resulting in an avalanche in the system.

The force exerted by the Maxwell elements in both SLS components can be defined as follows. Assume that *x* represents the total displacement of the Maxwell element and is defined by $x=x_{d}+x_{s}$, where $x_{d}$ is the displacement in the dashpot and $x_{s}$ is the displacement in the spring. It is possible to show that the force due to the Maxwell element satisfies the following ODE [39]:

(4) $$\frac{dF}{dt}=-k_{d}\frac{dx}{dt}-\frac{k_{d}}{\eta }F.$$

This was used to define the forces exerted by the Maxwell elements in the SLS component connected to the top plate, and on either side of block *i*, by blocks (i−1) and (i+1).

(5) $$\frac{dF_{\mathrm{Mp}}}{dt}=-k_{\mathrm{pd}}\frac{dx_{i}}{dt}-\frac{k_{\mathrm{pd}}}{\eta_{p}}F_{\mathrm{Mp}}\;,$$

(6) $$\frac{dF_{M}^{\left(i-1\right)}}{dt}=-k_{d}\frac{d(x_{i}-x_{i-1})}{dt}-\frac{k_{d}}{\eta }F_{M}^{\left(i-1\right)}\;,$$

(7) $$\frac{dF_{M}^{\left(i+11\right)}}{dt}=-k_{d}\frac{d(x_{i}-x_{i+1})}{dt}-\frac{k_{d}}{\eta }F_{M}^{\left(i+1\right)}\;.$$

Equations (6) and (7) can be combined into a single ordinary differential equation, where the force exerted by the Maxwell elements of both neighbor blocks is equal to $F_{M}=F_{M}^{\left(i-1\right)}+F_{M}^{\left(i+1\right)}$, and is defined by:

(8) $$\frac{dF_{M}}{dt}=-k_{d}\frac{d(2x_{i}-x_{i-1}-x_{i+1})}{dt}-\frac{k_{d}}{\eta }F_{M}\;.$$

A chain of *N* slider blocks can then be described by the system of ODE equations for each block *i*, which can then be solved numerically to simulate the motion of each block within a set time interval. The linearly elastic interactions between neighboring blocks and the upper plate result in an instantaneous transfer of stress within the system, while the presence of the viscous dashpots allows for a delay in transfer that enables the further potential triggering of slipping events [19,28,38,39].

Performing the nondimensionalization of this system of equations allows for a further analysis of the model behavior, while reducing the number of independent parameters. The following dimensionless variables can be introduced for this purpose:

(9) $$\tau =\frac{t}{\sqrt{\frac{m}{k_{\mathrm{pd}}}}}\;,\;u_{i}=\frac{x_{i}k_{\mathrm{pd}}}{f_{s}}\;,\;{\tilde{F}}_{M}=\frac{F_{M}k_{\mathrm{pd}}}{f_{s}k_{d}}\;,\;{\tilde{F}}_{\mathrm{Mp}}=\frac{F_{\mathrm{Mp}}}{f_{s}}\;,$$

where

(10) $$V_{i}={\dot{u}}_{i}=\frac{du_{i}}{d\tau }=\frac{\sqrt{mk_{\mathrm{pd}}}}{f_{s}}\frac{dx_{i}}{dt}=\frac{\sqrt{mk_{\mathrm{pd}}}}{f_{s}}{\dot{x}}_{i}=\frac{\sqrt{mk_{\mathrm{pd}}}}{f_{s}}v_{i}\;.$$

These nondimensional variables can be used to rewrite the equations of motion (3) with (5) and (8) in the following form:

(11) $$\begin{array}{ccc} \frac{d^{2}u_{i}}{d\tau^{2}} & = & -\omega \;\left(2u_{i}-u_{i-1}-u_{i+1}\right)-\omega_{p}\;u_{i}+\omega_{f}{\tilde{F}}_{M}\left(\tau \right)+{\tilde{F}}_{\mathrm{Mp}}\left(\tau \right)-\phi \left(z\right)\;,\\ \frac{d{\tilde{F}}_{M}}{d\tau } & = & -\frac{d(2u_{i}-u_{i-1}-u_{i+1})}{d\tau }-\omega_{d}\;{\tilde{F}}_{M}\;,\\ \frac{d{\tilde{F}}_{\mathrm{Mp}}}{d\tau } & = & -\frac{du_{i}}{d\tau }-\omega_{\mathrm{pd}}\;{\tilde{F}}_{\mathrm{Mp}}\;, \end{array}$$

where the new dimensionless parameters are given as follows:

(12) $$\begin{array}{c} \omega =\frac{k}{k_{\mathrm{pd}}},\;\omega_{p}=\frac{k_{p}}{k_{\mathrm{pd}}},\;\omega_{f}=\frac{k_{d}}{k_{\mathrm{pd}}},\\ \omega_{d}=\frac{k_{d}}{\eta }\sqrt{\frac{m}{k_{\mathrm{pd}}}},\;\omega_{\mathrm{pd}}=\frac{k_{\mathrm{pd}}}{\eta_{p}}\sqrt{\frac{m}{k_{\mathrm{pd}}}}\;. \end{array}$$

The parameters ω and $\omega_{p}$ describe the elastic coupling of the system, and $\omega_{f}$, $\omega_{pd}$, and $\omega_{d}$ dictate the viscous response of the SLS elements. The variable representing the velocity of each block *i* within the velocity-weakening friction law, *z*, is given by

(13) $$z=\frac{v_{i}+v_{\mathrm{dr}}}{v_{0}}=\delta \;\left(V_{i}+\nu \right),$$

where

(14) $$\delta =\frac{1}{v_{0}}\frac{B}{A}=\frac{1}{v_{0}}\frac{f_{s}}{k_{\mathrm{pd}}}\sqrt{\frac{k_{\mathrm{pd}}}{m}}\;,\;\nu =v_{\mathrm{dr}}\frac{k_{\mathrm{pd}}}{f_{s}}\sqrt{\frac{m}{k_{\mathrm{pd}}}}\;.$$

To simulate the model using this system of equations, one can use the following switch algorithm, which allows for transitions between the stick and slip states [40,41]. This is evaluated at each time step within the numerical solution process, where $F_{\mathrm{SLS}}^{\left(i\right)}$ is the force applied by both the upper plate and neighboring blocks (Algorithm 1).
**Algorithm 1:** The switch algorithm to simulate the numerical integration of slider blocks assembled in a chain.1:**procedure** SliderBlock($u_{i},u_{i-1},u_{i+1},V_{i},V_{i-1},V_{i+1},\omega ,\omega_{p},\omega_{f},\omega_{d},\omega_{\mathrm{pd}},\nu ,dV$)2:    $F_{\mathrm{SLS}}^{\left(i\right)}\leftarrow -\omega \;\left(2u_{i}-u_{i-1}-u_{i+1}\right)-\omega_{p}\;u_{i}+\omega_{f}{\tilde{F}}_{M}\left(\tau \right)+{\tilde{F}}_{\mathrm{Mp}}\left(\tau \right)$  ▹ compute the force3:    **if** $\left|V_{i}+\nu \right|>dV$**then**                        ▹ slip phase4:        $\frac{du_{i}}{d\tau }\leftarrow V_{i}$5:        $\frac{dV_{i}}{d\tau }\leftarrow F_{\mathrm{SLS}}^{\left(i\right)}-\phi \left(z\right)$6:    **else if** $\left|F_{\mathrm{SLS}}^{\left(i\right)}\right|>1$ **then**                   ▹ stick to slip transition7:        $\frac{du_{i}}{d\tau }\leftarrow V_{i}$8:        $\frac{dV_{i}}{d\tau }\leftarrow F_{\mathrm{SLS}}^{\left(i\right)}-\mathrm{sign}\left(F_{\mathrm{SLS}}^{\left(i\right)}\right)$9:    **else**                               ▹ stick phase10:        $\frac{du_{i}}{d\tau }\leftarrow -\nu$11:        $\frac{dV_{i}}{d\tau }\leftarrow -\left(V_{i}+\nu \right)$12:    $\frac{d{\tilde{F}}_{M}}{d\tau }\leftarrow -\left(2V_{i}-V_{i-1}-V_{i+1}\right)-\omega_{d}\;{\tilde{F}}_{M}$13:    $\frac{d{\tilde{F}}_{\mathrm{Mp}}}{d\tau }\leftarrow -V_{i}-\omega_{\mathrm{pd}}\;{\tilde{F}}_{\mathrm{Mp}}$14:    **return** $\left(\frac{du_{i}}{d\tau },\;\frac{dV_{i}}{d\tau },\;\frac{d{\tilde{F}}_{M}}{d\tau },\;\frac{d{\tilde{F}}_{\mathrm{Mp}}}{d\tau }\right)$          ▹ return the slider block state

Finally, for the model to produce suitable results, boundary conditions must be assigned to prevent irregular behavior on either side of the slider-block chain. For this model, the displacement of the end blocks was set to zero. Additionally, the initial condition for each slider block is the “stuck” position, with a velocity of zero relative to the conveyor belt. To provide the required spatial heterogeneity in the model, the initial displacement of each block was generated according to uniformly distributed random numbers within a limited interval.

Within the simulation itself, an analysis was performed, following an initial transient regime to allow for the system to fall into a statistically steady state. During the steady state, a procedure was conducted to identify, examine, and record the properties of the observed block movement that form clusters or avalanches. The properties of each cluster, including the number of involved blocks, the velocity at which the slippage events occurred, and the area over which the total displacement occurred, were used to quantify the size of each avalanche.

It is the size and frequency regarding these events that form the basis of the following analysis. Particularly, the triggering and decay of avalanche events following a sufficiently large event was analyzed using the ETAS model [25]. This model describes aftershock sequences as a clustering of seismic activity; each earthquake triggers a subsequent proportional increase in the rate of earthquake events depending on the frequency and magnitude of past earthquakes. The variation of event occurrence rate can be described by the following equation [25]:

(15) $$\lambda_{\omega }\left(t\right)=\mu +K\sum_{i:t_{i}<t}^{N_{t}}\frac{e^{\alpha (m_{i}-m_{0})}}{{\left(\frac{t-t_{i}}{c}+1\right)}^{p}}\;,$$

where $\lambda_{\omega }\left(t\right)$ is the event rate with respect to time, with reference magnitude $m_{0}$ and the model parameters ω={μ,α,K,c,p}. In this model, the rate is a superposition of a constant background activity rate μ, alongside contributions from each previous event. The parameter *c* describes the rate of aftershocks in the beginning stages of an aftershock sequence, the parameter *p* describes the speed at which the aftershock rate decays, and both the parameters *K* and α describe the productivity of an aftershock sequence.

The parameters of simulated aftershock sequences according to the ETAS model were estimated using the maximum likelihood estimation (MLE) method, utilizing the corresponding log-likelihood function:

(16) $$\begin{array}{c} \log\left(L\right)=-\mu \left(T_{e}-T_{s}\right)-\frac{Kc}{p-1}\sum_{i=1}^{k}e^{\alpha (m_{i}-m_{0})}\left[{\left(\frac{T_{s}-t_{i}}{c}+1\right)}^{1-p}-{\left(\frac{T_{e}-t_{i}}{c}+1\right)}^{1-p}\right]\\ -\frac{Kc}{p-1}\sum_{i=k+1:T_{s}\leq t_{i}\leq T_{e}}^{N_{T_{e}}}e^{\alpha (m_{i}-m_{0})}\left[1-{\left(\frac{T_{e}-t_{i}}{c}+1\right)}^{1-p}\right]\\ +\sum_{j=1}^{n}\log\left[\mu +K\sum_{i:t_{i}<t_{k+j}}^{N_{t_{k+j}}}\frac{e^{\alpha (m_{i}-m_{0})}}{{\left(\frac{t_{k+j}-t_{i}}{c}+1\right)}^{p}}\right]\;, \end{array}$$

where $t_{i}$ is the event times within a time interval $\left[T_{0},T_{e}\right]$ in a given catalog with $N_{T_{e}}$ events and the time interval $\left[T_{s},T_{e}\right]$, with $T_{s}>T_{0}$ encompassing all events within the fitting time interval. *k* is the number of events in the interval $\left[T_{0},T_{s}\right]$ and *n* is the number of event in $\left[T_{s},T_{e}\right]$.

## 3. Results

### 3.1. Model Simulation

Simulations of the above viscoelastic slider-block model were performed through the numerical integration of a system of ODE equations for a linear array of N=100 slider blocks. Each simulation was permitted to complete an initial transient regime, after which statistics and observations were collected regarding the behavior of the system while in a steady state. Each simulation had a transient regime length of τ=10,000, followed by a steady state regime with length τ=50,000.

The simulation described in this section is characterized by the dimensionless model parameters $\left\{\omega ,\omega_{p},\omega_{f},\omega_{d},\omega_{\mathrm{pd}},\nu ,\delta \right\}=\left\{8,2.5,1.5,0.03,0.01,0.001,10\right\}$. The degree of elastic coupling within the system is defined by the initial three parameters, while parameters $\omega_{d}$ and $\omega_{\mathrm{pd}}$ (12) define the magnitude of the viscous response of the SLS components connected between slider blocks and to the upper plate, respectively. The magnitude of the friction between slider blocks and the conveyor belt is determined by the parameter δ (14), and the driving velocity of the conveyor belt is determined by the parameter ν (14).

These dimensionless parameters $\left\{\omega ,\omega_{p},\omega_{f},\omega_{d},\omega_{\mathrm{pd}},\nu ,\delta \right\}$ define the amplitudes of the corresponding forces acting on each slider block. Values were chosen to reproduce realistic behaviour observed for natural seismicity, and later on, these values were varied to enhance the viscoelastic effect, and to observe how the frequency–magnitude behaviour changes accordingly. For this, we used small values of $\omega_{p}$ and $\omega_{pd}$, as they are entered as a dumping parameter for forces $F_{M}$ and $F_{\mathrm{Mp}}$ in (11). The smaller values result in longer viscous effects associated with the slippage of each block. Parameter ν was chosen to be small as it represents the driving or loading velocity.

**Figure 2:**
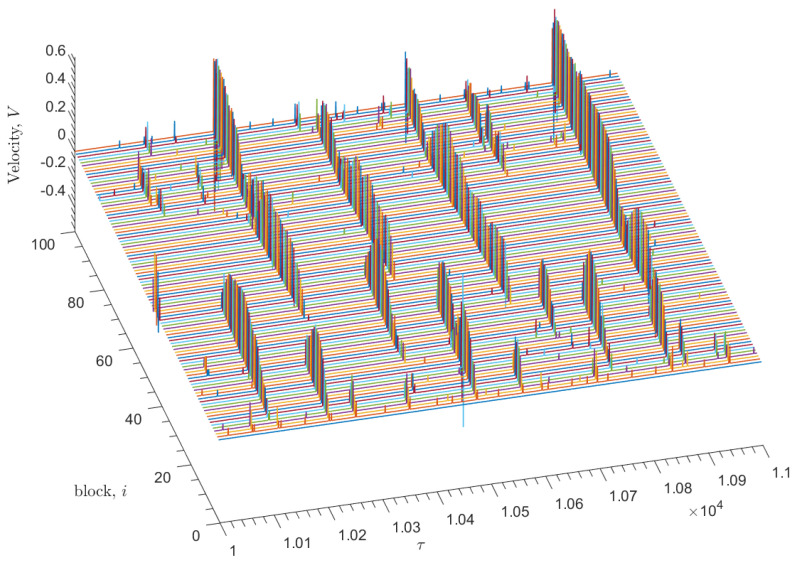
Nondimensional steady-state velocity measurements with time τ for N=100 slider blocks for the simulation of the model with parameters ω=8, $\omega_{p}=2.5$, $\omega_{f}=1.5$, $\omega_{d}=0.03$, $\omega_{\mathrm{pd}}=0.01$, ν=0.001, δ=10, recorded following an initial transient regime. Sharp velocity spikes spanning multiple slider blocks within a sufficiently small time interval are counted as avalanche events. Velocity is measured in arbitrary units.

**Figure 3:**
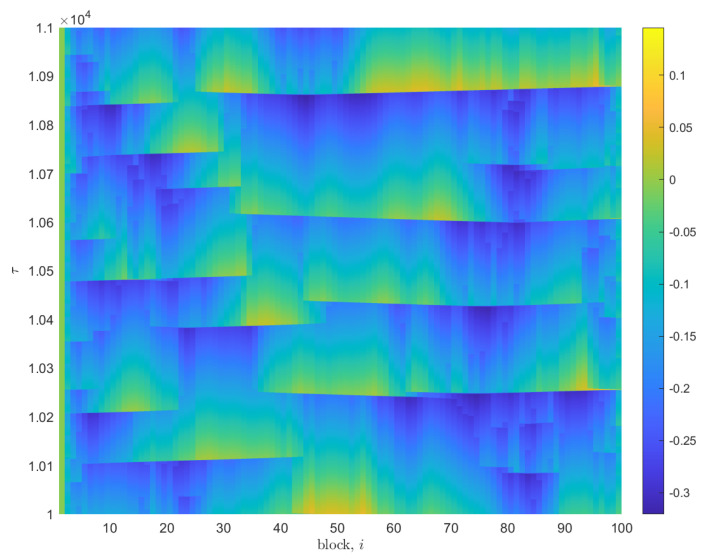
Pseudocolor plot displaying nondimensional displacement measurements with time τ, for N=100 slider blocks, for an iteration of the model with the same parameters as in Figure 2. The magnitude of displacement is represented by the adjacent color bar, with arbitrary units.

**Figure 4:**
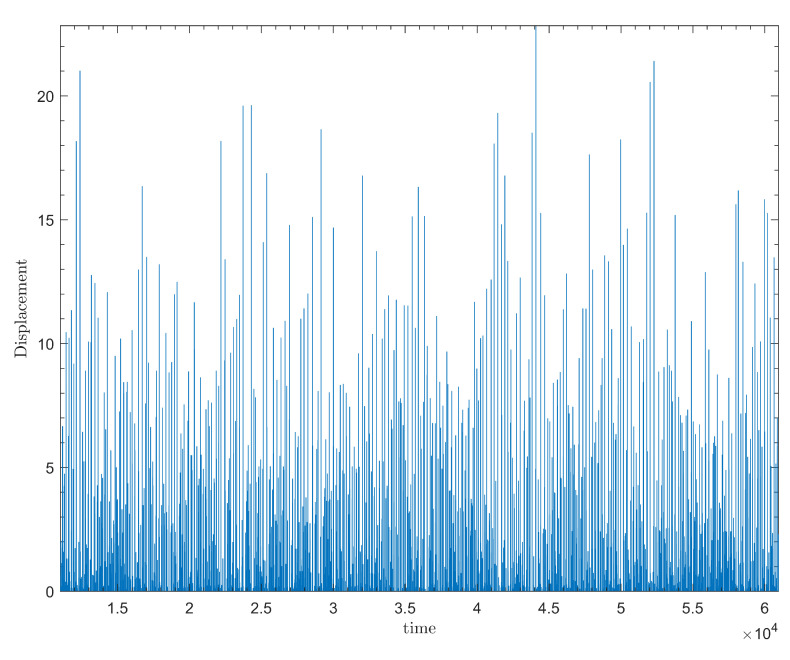
Plot depicting the evolution of event sizes in time for N=100 slider blocks, and for the simulation of the model with the same parameters as in Figure 2.

The velocity of each slider block $v_{i}$, determined at each time step of the numerical solution, is shown in Figure 2. The sharp, velocity spikes represent the sudden slippage of a slider block that occurs once the nonlinear friction force is overcome. Sufficiently large sudden displacements of a single slider block acts as a trigger for neighboring blocks to undergo similar displacements, generating an avalanche. The size, *s*, of each avalanche is determined by the sum displacement of each block involved in the avalanche. Figure 3 displays the displacement resulting from the above velocity spikes. Figure 4 displays the distribution of these events in time.

The simulation collects characteristics of the avalanches, including the block at which the sequence was initiated and the duration of the avalanche, as defined by the period of time in which any block within the event has a velocity relative to the conveyor belt greater than some small cutoff value. This cutoff value allows for a distinction between avalanche events. In both Figure 2 and Figure 3, regular instances of both negative velocity and negative or “backward” displacement may be observed alongside movement in the positive direction.

For the model simulations performed in this work, a single run with the specified transient and steady time intervals took several hours to run on a PC computer. Increasing the system size can be a challenging task; however, the implementation of the parallel version of the model can help to speed up the computations.

### 3.2. Avalanche Statistics and Model Fitting

**Figure 5:**
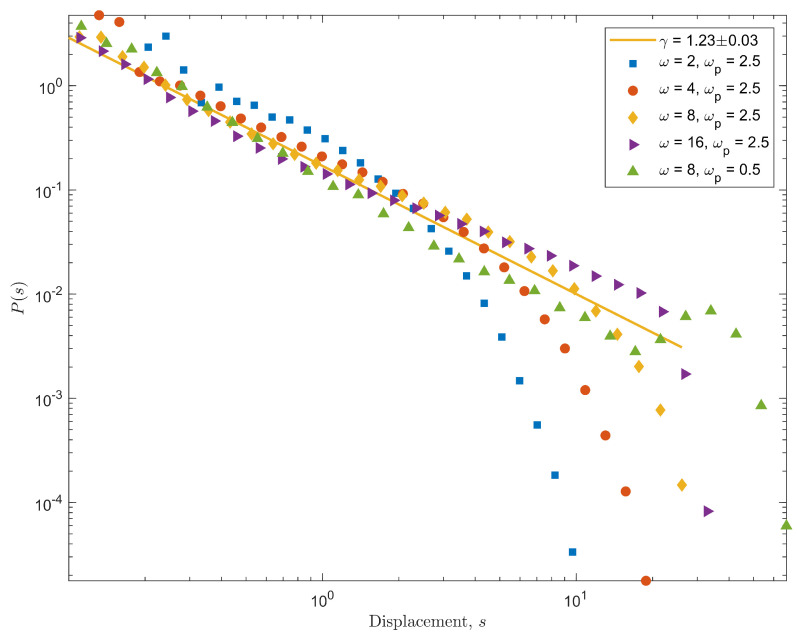
Frequency-size distribution of simulated avalanches for the simulations of the model with N=100 blocks, with parameters $\omega_{f}=1.5$, $\omega_{d}=0.03$, $\omega_{\mathrm{pd}}=0.01$, ν=0.001, δ=10, and varying parameters ω and $\omega_{p}$. The corresponding parameters for each simulation run are in the legend, with the associated symbol. The straight solid line corresponds to the power–law fit $P\left(s\right)∼s^{-\gamma }$ to the data with ω=8 and $\omega_{p}=2.5$.

The viscoelastic slider-block model was capable of generating frequency-size distributions, resembling those observed in natural seismicity [21,22,42]. The frequency-size distributions associated with five parameter variations can be observed in Figure 5, alongside an associated power–law fit $P\left(s\right)∼s^{-\gamma }$, where P(s) is the probability distribution function for the sum displacement *s*, and γ is the scaling exponent. Each simulation was performed over τ=200,000, following an initial transient regime.

**Figure 6:**
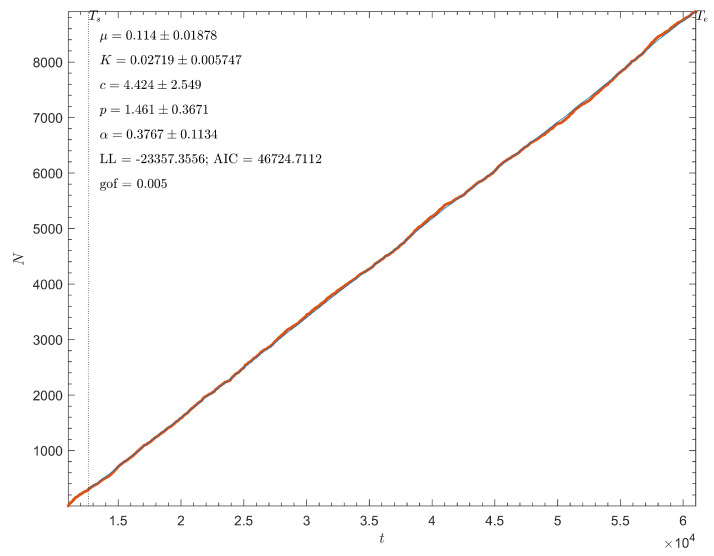
ETAS model fitting for cumulative simulated avalanche events for an iteration of an N=100 linear array, with parameters $\omega =8,\;\omega_{p}=2.5,\;\omega_{f}=1.5,\;\omega_{d}=0.03,\;\omega_{\mathrm{pd}}=0.01,\;\nu =0.001,\;\delta =10$. The red points represent the cumulative number of events during the given nondimensional time interval. The blue line represents the corresponding ETAS fit using MLE (15). All parameters are displayed within a 95% confidence interval.

When compared to naturally occurring aftershock sequences, the temporal clustering observed in this model deviated slightly. The slider-block avalanche sequences were described using parameters obtained from the fitting of the ETAS model. The ETAS model is used to describe the rate of aftershock generation, Equation (15), as a direct response to the triggering and decay of seismic activity following prior earthquake events. Parameters were obtained using maximum likelihood estimation. A comparison between the cumulative number of simulated avalanche events for one simulation with the given model parameters during a specific time interval and the corresponding ETAS fit is shown in Figure 6.

As the model avalanches do allow for a successful ETAS model fitting, temporal clustering does exist within the model; however, across repeated simulations, the estimated *p*-value took a value of approximately 1.46, and an α-value of 0.377. These values, respectively, indicate that the aftershock rate of decay is relatively high, and that the aftershock rate stays relatively constant during this time interval, then drops abruptly. When reviewed in contrast to Omori’s law, this behavior indicates that these simulated aftershock sequences may not be as prolific as naturally occurring aftershock sequences. This α-value is lower than typically observed in real-life earthquake events, indicating that generated aftershock sequences are not as vigorous as those observed in real world; however, a *p*-value of 1.46 is a physically reasonable value, and indicates similarity between simulated and real earthquake behavior [43,44].

## 4. Discussion and Conclusions

The objective of this work was to use the viscoelastic slider-block model to replicate the conditions of a seismogenic fault within a medium similar to that of the Earth’s crust. This model is characterized by the introduction of SLS elements, and subsequently, the introduction of nonlinear viscous processes involved with the redistribution of stress. Simulations were performed to determine the role of viscous processes in the generation of avalanche events that follow well-known laws regarding frequency-size distribution and aftershock decay rates.

The simulations provided favorable results regarding frequency-size distributions, resulting in consistent power–law scaling [45,46,47]. All simulations reproduced frequency-size relations similar to those generated by natural earthquake dynamics. Using this model, and other similar slider-block models exhibiting elements of self-organized criticality, simulations can produce realistic results and can re-affirm the hypothesis that naturally occurring frequency-size distributions of events may partially be the result of SOC within the Earth’s crust [9,31].

Moreover, the ETAS model fitting produced favorable results, despite relatively high *p*-values and a low productivity of aftershock sequences reflected in the α parameter. Using the input model parameters outlined in the previous section, model fitting returned *p*-values of approximately 1.46, demonstrating a comparatively high, but still physically reasonable, decay rate of aftershocks. The results of this work confirm that to observe an Omori-like decay rates for aftershock sequences, one needs to consider the viscous effects governed by linear or power–law rheology as was demonstrated by similar slider-block and cellular automata models [19,29,30].

In this paper, initial conditions and physical parameters were varied to observe how frequency–magnitude statistics change as the properties of the model change. In future works, a full analysis of the parameter space of the model will be completed, both to determine the effects of the input parameters on the frequency–magnitude distribution, but also to determine the relationship between input parameters and corresponding ETAS model parameters. This will help deduce the physical meaning of the ETAS parameters, and may provide a theoretical basis to support the application of the ETAS model to natural seismicity. In particular, future model parameter space exploration should further examine how variations in viscoelasticity influence the generation of simulated earthquake sequences.

The ETAS model describes the behavior of aftershock sequences as a direct consequence of previous seismic activity. This places an emphasis on the redistribution of stress within a system following any seismic activity, as events are generated not through the direct constant application of force (plate tectonics, conveyor belt), but through previous trigger activity and instability [28,48]. The introduction of nonlinear behavior within a medium provides a mechanism through which this redistribution of stress may be delayed, allowing for the generation of aftershocks. The presence of temporal clustering within the system described by this model supports the theory that nonlinear viscoelasticity influences the generation of aftershocks, and that viscous responses within the Earth’s crust and upper mantle may contribute to the observed aftershock dynamics.

## Data Availability

No new data were created in this work.
